# Dynamics of the Bloch point in an asymmetric permalloy disk

**DOI:** 10.1038/s41467-019-08327-6

**Published:** 2019-02-05

**Authors:** Mi-Young Im, Hee-Sung Han, Min-Seung Jung, Young-Sang Yu, Sooseok Lee, Seongsoo Yoon, Weilun Chao, Peter Fischer, Jung-Il Hong, Ki-Suk Lee

**Affiliations:** 1 Center for X-ray Optics, Lawrence Berkeley National Laboratory, Berkeley, CA 94720 USA; 2 Department of Emerging Materials Science, DGIST, Daegu, 42988 Republic of Korea; 3 School of Materials Science and Engineering, Ulsan National Institute of Science and Technology, Ulsan, 44919 Republic of Korea; 4 Advanced Light Source, Lawrence Berkeley National Laboratory, Berkeley, CA 94720 USA; 5 Materials Sciences Division, Lawrence Berkeley National Laboratory, Berkeley, CA 94720 USA; 6 Department of Physics, University of California, Santa Cruz, Santa Cruz, CA 94056 USA

## Abstract

A Bloch point (BP) is a topological defect in a ferromagnet at which the local magnetization vanishes. With the difficulty of generating a stable BP in magnetic nanostructures, the intrinsic nature of a BP and its dynamic behaviour has not been verified experimentally. We report a realization of steady-state BPs embedded in deformed magnetic vortex cores in asymmetrically shaped Ni_80_Fe_20_ nanodisks. Time-resolved nanoscale magnetic X-ray imaging combined with micromagnetic simulation shows detailed dynamic character of BPs, revealing rigid and limited lateral movements under magnetic field pulses as well as its crucial role in vortex-core dynamics. Direct visualizations of magnetic structures disclose the unique dynamical feature of a BP as an atomic scale discrete spin texture and allude its influence on the neighbouring spin structures such as magnetic vortices.

## Abstract (web summary)

The intrinsic nature and dynamics of a Bloch point has not been verified so far. Here, Im et al. report the realization and dynamical character of steady-state Bloch points in the magnetic vortex cores in asymmetrically shaped Ni_80_Fe_20_ nanodisks.

## Introduction

Topological singularities have been a fascinating concept in physics playing crucial roles in a variety of non-trivial physical phenomena such as quantum phase transition, skyrmion crystals, and superfluid ^3^He^1–5^. One example in magnetism is the point-like topological defect referred as the Bloch point (BP), which was originally proposed by Feldtkeller^6^ and Döring^7^.

**Fig. 1:**
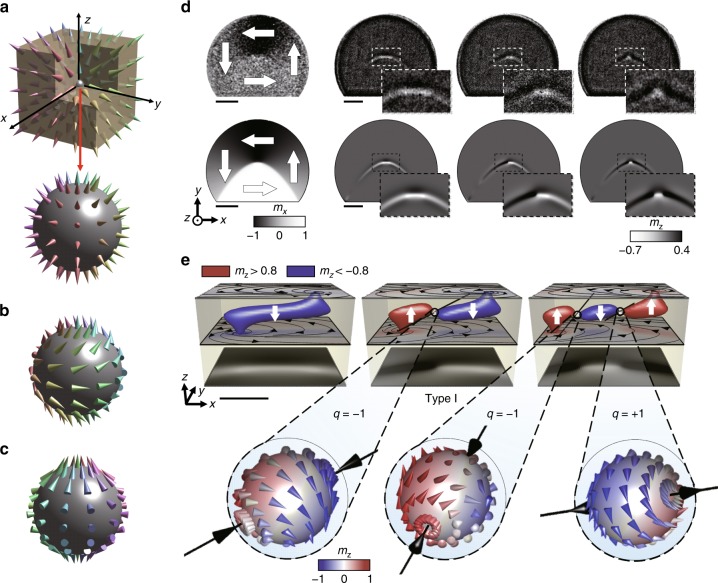
Direct observation of stabilized Bloch point (BP) structures. **a**–**c** Schematic diagram of three possible configurations of a BP with skyrmion charge *q* = + 1, namely, hedgehog (**a**), circulating (**b**), and spiralling (**c**) configurations where the colour indicates the orientation of magnetization. **d** In-plane (IP) and out-of-plane (OOP) magnetic components observed by magnetic transmission soft X-ray microscopy (MTXM), and the corresponding magnetic structures determined by micromagnetic simulations. Zoomed images for magnetic configurations near vortex core structures are also inserted. The black and white contrasts in the images of the IP (OOP) magnetic component represent the magnetizations oriented in the left and right directions on the disk plane (perpendicular upward and downward to the disk plane), respectively. **e** Images of the distorted vortex core structures with no BP (left), a single BP (middle), and double BPs (right), and the configurations of the BPs embedded in the vortex cores. The OOP magnetic components with *m*_z _>  + 0.8 (red) and *m*_z_ < −0.8 (blue) were extracted and the BP configurations were obtained by interpolation from the simulated vortex core structures. The single BP is characterized by *q* = −1, while the double BPs are defined by *q* = −1 and +1, respectively. The single-BP core structure here is referred to as type I. Scale bars in (**d**, **e**) correspond to 500 nm and 50 nm, respectively

A BP is characterized by the skyrmion charge *q*:

$$q = 1/8\pi {\int} {\mathrm{d}}A_{\it i}\varepsilon _{\it ijk}\widehat {\bf{m}} \cdot \partial _{\it j}\widehat {\bf{m}} \times \partial _{\it k}\widehat {\bf{m}} = \pm 1,$$

where $\widehat {\bf{m}}$ is the unit vector of magnetization, and the integration is taken over a closed surface *A* surrounding the BP. There are three main possible configurations of a BP (Fig. 1a–c): namely, the hedgehog configuration, in which the distribution of the magnetization around the BP is spherically symmetric and the magnetization vectors are oriented away from the BP (*q* = +1) or toward the BP (*q* = −1), and circulating (*q* = +1 or −1) and spiral (*q* = +1 or −1) configurations, which are obtained by 90° and 180° global rotations of the magnetic moments of the hedgehog configuration, respectively^8–10^. In contrast to other topological spin textures such as magnetic skyrmions and vortices, the BP has a unique feature—the local magnetization at a BP completely vanishes. The existence of BP structures has been confirmed in yttrium iron garnet crystal plates, micrometre-thick garnet films, and magnetic cylindrical wire based on static measurements^11–13^.

In recent years, BPs are attracting more attention, particularly in nanomagnetic systems, since many theoretical works carried out in dynamic conditions have proposed that BPs bear critical roles in many dynamical phenomena of spin textures and influence their equilibrium states. For example, it was discussed that BPs work as key components to initiate the magnetization reversal process and domain wall (DW) motion in the magnetic cylinders^14–18^. They are also known to mediate the switching of vortex and skyrmion cores^19–24^. Injection and/or expulsion of BPs during the motion of magnetic DWs and switching of magnetic vortices or skyrmions were reported to change the topological numbers in spin textures^1,21,25,26^.

Even though extensive theoretical and experimental studies have been conducted on topological spin textures in confined magnetic elements^27–31^, understanding of the underlying physics on BP dynamical behaviours and their influences on the overall dynamics of nanomagnetic system in response to the perturbations is still missing. Due to the limitations in achieving both temporal and spatial resolutions simultaneously to capture the dynamic motions of BPs, experimental attempts have been focused on observing the static state of a BP and its quasi-static motions^13,16,32^. Thus, experimental investigation of BP dynamics and related phenomena in nanopatterned magnetic systems have never been fully addressed. In a confined magnetic nanoscale system, another primary challenge hindering the study of BP dynamics is the difficult generation of steady-state BPs without the assistance of external source such as magnetic fields. In the present work, we generate steady-state BPs in association with magnetic vortex cores of asymmetrically shaped 100 nm thick permalloy (Ni_80_Fe_20_, Py) disks and their dynamic behaviours are directly observed along with their quasi-static motions using the magnetic transmission soft X-ray microscopy (MTXM) at the Advanced Light Source with a spatiotemporal resolution of 25 nm and 70 ps^33^.

## Results

### X-ray imaging of the BP core structure

Figure 1d shows the MTXM images of the in-plane (IP) and out-of-plane (OOP) magnetic components observed in a Py disk of height *h* = 100 nm, diameter *D* = 2.5 µm, and asymmetry ratio *r* = 0.2*D* in direct comparison with those obtained by micromagnetic simulations. Asymmetric disks were chosen with the consideration of previous studies reporting that the vortex core deforms as an elongated tube-shaped DW in asymmetric magnetic patterns with its conserved topology^34,35^ and if there is a transition of the DW structure, particularly core switch alone case^36^, a topological spin texture of an integer skyrmion number such as a BP, *q* = + 1 (−1) might be added to keep the total skyrmion number of the system, *q* = 0.

When the disk was repeatedly saturated to +1 kOe and released to the remanent state, counterclockwise circularity (CCW, *c* = −1) of the IP magnetic components was created most of the time, while three different types of OOP magnetic components were observed. It should be noted that the OOP magnetic components observed in the disk are not circular but exhibit quite deformed state in the asymmetric disk^34,35^. Instead of point-like vortex core with either upward or downward magnetization at the centre of the disk^29^, as usually observed in a fully circular magnetic element, the core is extended in the form of an arc-shaped line with OOP magnetizations. Moreover, the magnetizations within the OOP magnetic components are not always aligned in the same direction, but are divided into two parts with oppositely directed OOP magnetizations. Matching IP and OOP magnetization images were reproduced by micromagnetic simulations performed in the disk with identical geometry (Fig. 1d). Zoomed images of OOP magnetic components inserted in Fig. 1d shows that the MTXM images and images taken from micromagnetic simulations are matching with excellent consistency (see Methods for details).

Since the MTXM measurement was performed with two-dimensional transmission imaging methods, detailed OOP components at the centre of the disk along the *z*-axis were illustrated from the simulated magnetic configurations (Fig. 1e). The voxels with strong *z*-components of the magnetizations (*m*_*z*_ > + 0.8 and *m*_*z*_ < −0.8) are grouped as blue and red, respectively. The elongated OOP magnetic component observed in the MTXM measurements were identified to be vortex cores noncoaxially located at the top and bottom surfaces of the disk. Such deformed vortex core structures are energetically favourable due to the asymmetrically shaped disk’s facet combined with the disk height of *h* = 100 nm^37^. The tube-shaped magnetic segment in Fig. 1e that connects the vortex cores at the top surface and bottom surface was confirmed to be an asymmetric Bloch wall (ABW), consisting of OOP Bloch components in the middle part along the disk thickness and IP magnetic components on the disk surface^34–36,38,39^. The ABW formed a flux-closure domain (FCD) in which the Néel caps on the two surfaces point in opposite directions^36,39,40^ (see Supplementary Figure 1 and Supplementary Note 1). Unlike the structure of the first core, the second (third) core structure in Fig. 1e includes a point at which the orientation of the magnetization flips with the vortex cores on the top surface and bottom surface pointing in opposite directions (vortex cores oriented in the same direction with the Bloch wall segment pointing in opposite directions). Remarkably, at the point where magnetization orientation switches, the local magnetization was found to completely vanish (*m*_*x* _= *m*_*y*_ = *m*_*z*_ = 0) (see Supplementary Figure 2 and Supplementary Note 2), which is regarded as the signature for magnetic singularity, namely, the BP. Whereas the first core in Fig. 1e contains no BP, hence referred to as the non-BP core, the second and third cores hold single BP and double BPs, and are hereafter referred to as the single-BP and double-BP cores, respectively. It is notable that overall shape including the curvature of deformed vortex core becomes different as BPs present, which is clearly shown in the zoomed insets in Fig. 1d. In order to confirm the existence of BPs in observed magnetic configurations, we strictly surveyed similarly looking magnetic structures where OOP magnetic components having opposite polarizations coexist including asymmetric Néel walls and vortex–antivortex–vortex structures. All of them, except the proposed magnetic vortex structures with associated BPs, are energetically unstable that they cannot be established in the asymmetric disk.

Complete spin configurations around the BPs were obtained by interpolation, as also shown in Fig. 1e. Arrows indicate the unit vectors of the magnetization over the closed spherical surface at a distance of 2 nm from the BP. The magnetization rotates along the central axis near the equator, with the magnetic vectors tending to tilt toward the poles with increasing latitude, which is consistent with the configuration of a circulating BP^8–10^. The BP in the single-BP core exhibits *q* = −1 as the magnetization along the central axis points toward the BP, and the double-BP core holds the other BP characterized by *q* = + 1 in addition to the BP of *q* = −1. The total energy of a non-BP core (2.48 × 10^−16^ J) is found to be lower than the single-BP core (2.52 × 10^-−16^ J) and the double-BP core (2.57 × 10^−16^ J), indicating that BP cores are metastable states. Through the 30 repeated measurements for the formation of vortex cores in 5 different disks with identical dimensions (*h* = 100 nm, *D* = 2.5 µm, and *r* = 0.2*D*), which leads to a high statistical ensemble of 150, we indeed confirmed that the non-BP core is more favourably formed within the repetitions. This result supports that the BP cores are metastable states as expected from the calculations of magnetic energies. On the other hand, unlike the total energy, the demagnetization energies of the single-BP core (1.04 × 10^−16^ J) and double-BP core (1.02 × 10^−16^ J) were calculated to be slightly lower than that of the non-BP core (1.06 × 10^−16^ J). The magnetic surface charge was also confirmed to decrease with the formation of BPs in the system as reported in the previous study^9^, implying that the demagnetization energy is reduced when BPs are present. Therefore, BPs can form to reduce the demagnetization energy, and it explains why BPs are created although BP cores are in higher energy states than that of non-BP core. Energy densities and differences of all magnetic energies, anisotropy, exchange, demagnetization, and total energies among three core structures are provided in the supplementary data (see Supplementary Table 1, Supplementary Figures 3, 4 and Supplementary Note 3).

Another notable observation from Fig. 1e is the asymmetric volumes of the up-core and down-core in the single-BP core. The volume of down-core on the right side of the BP is obviously larger than the other, and the BP is slightly pushed in −*x*-direction toward the smaller core segment (type I). This is also evidently observed in the experimental image measured by MTXM (Fig. 1d).

### Different types of single-BP core structures

**Fig. 2:**
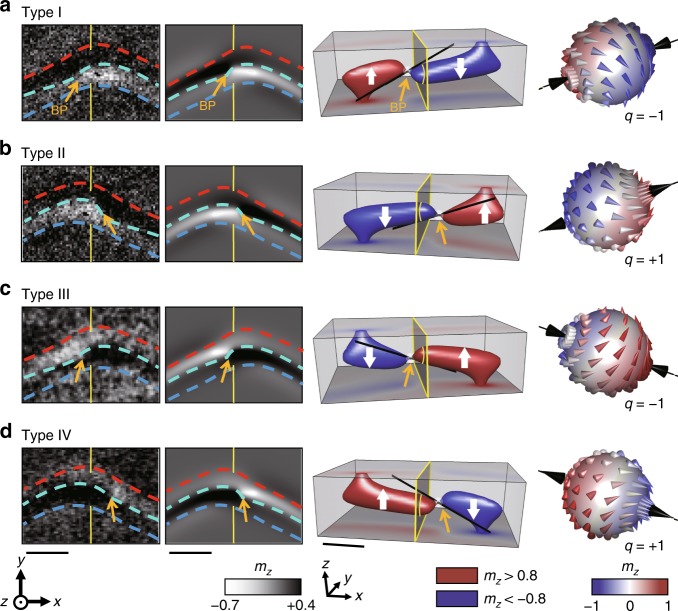
Different types of single-Bloch point (BP) core structures. **a**–**d** Single-BP core structures with different magnetic configurations, referred to as type I (**a**), type II (**b**), type III (**c**), and type IV (**d**). The zoomed-in magnetic transmission soft X-ray microscopy (MTXM) images for BP core structures together with simulated images are shown in the first and the second columns (scale bars: 150 nm), respectively. The boundaries between the black and white contrasts are outlined by the red, turquoise, and blue dotted lines. The simulated internal structures are shown in the third column (scale bar: 50 nm), and the configurations of the BPs contained in the vortex core structures are shown in the fourth column. The yellow line outline the central plane in the disk. The categorization is based on the polarizations of the cores on the top surface and bottom surface of the disk, volumes of cores, and the location of the BP

Within the repeated experiments for vortex formation in the same disk with identical field sequence from *H*_*x*_ = + 1 kOe to 0 Oe, three other equivalent structures were further identified in the single-BP cores, looking slightly different from the one in Fig. 1d, as shown in Fig. 2. Zoomed images for magnetic configurations around the core structures taken from MTXM measurements and micromagnetic simulations are displayed together so that the differences in detailed magnetic configurations of the four degenerate states are clearly recognized. The three-dimensional internal structures and BP configurations of the four degenerated structures, as determined by micromagnetic simulations, are also included in Fig. 2. The yellow plane in the figure indicates the central plane in the disk located at an equal distance from the planar positions of two vortex cores at the top and bottom surfaces of the disk. Note that the rotational sense of the circularity was the same CCW in all measurements.

The structure in Fig. 2b consists of a down-core with a larger volume as observed in the type I structure in Fig. 2a, although it is on the left side of the BP in the present case. The BP is shifted in the +*x*-direction from the centre of the disk (type II). The BP configuration enclosed in such a structure is characterized by *q* = + 1 with the magnetizations along the central axis pointing away from the BP. Contrary to the type I and II structures, the structures in Fig. 2c (type III) and Fig. 2d (type IV) include an up-core with a volume larger than that of the down-core, and the BP can accordingly be located on either the left (Fig. 2c) or right (Fig. 2d) side of the disk centre. The BPs in these structures are characterized with *q* = −1 and +1, respectively.

Energy calculations reveal that magnetic energies of all the four single-BP core structures above are equivalent with less than 0.004% differences among them (see Supplementary Table 2, Supplementary Figures 5, 6 and Supplementary Note 4). The degeneracy of these four different BP core structures, categorized as circulating BP configurations, is rather surprising because two energetically degenerate BP structures have been expected to exist in a circulating BP configuration^8–10^. The existence of the fourfold degenerate states in the asymmetric disk was found to be related with a symmetry breaking in the formation of FCD along with the ABW in the disk (see Supplementary Figure 5 and Supplementary Note 4). Another interesting point derived from the symmetry breaking in the formation of FCDs is that ABWs in those single-BP core structures only have polarization switch without Néel cap switch, which is analogous to the core switch alone case in DW transitions^36,41^. The rotation senses of FCDs created for ABW segments are identical and it means that the Néel caps of the two ABW segments head into the same direction on one disk surface. The outlines of OOP magnetic components of FCDs paired with ABW segments for each type of the single circulating BP core in zoomed images (Fig. 2) indicate the rotation senses of FCDs. This is also a unique feature of ABWs formed in the asymmetric disk. In symmetric magnetic disks such as rectangular pattern and/or thin films, DW transitions of all three cases, core switch alone, cap switch alone, and both core switch and cap switch can be created^36,41^.

### Quasi-static field-driven motion of the BP cores

**Fig. 3:**
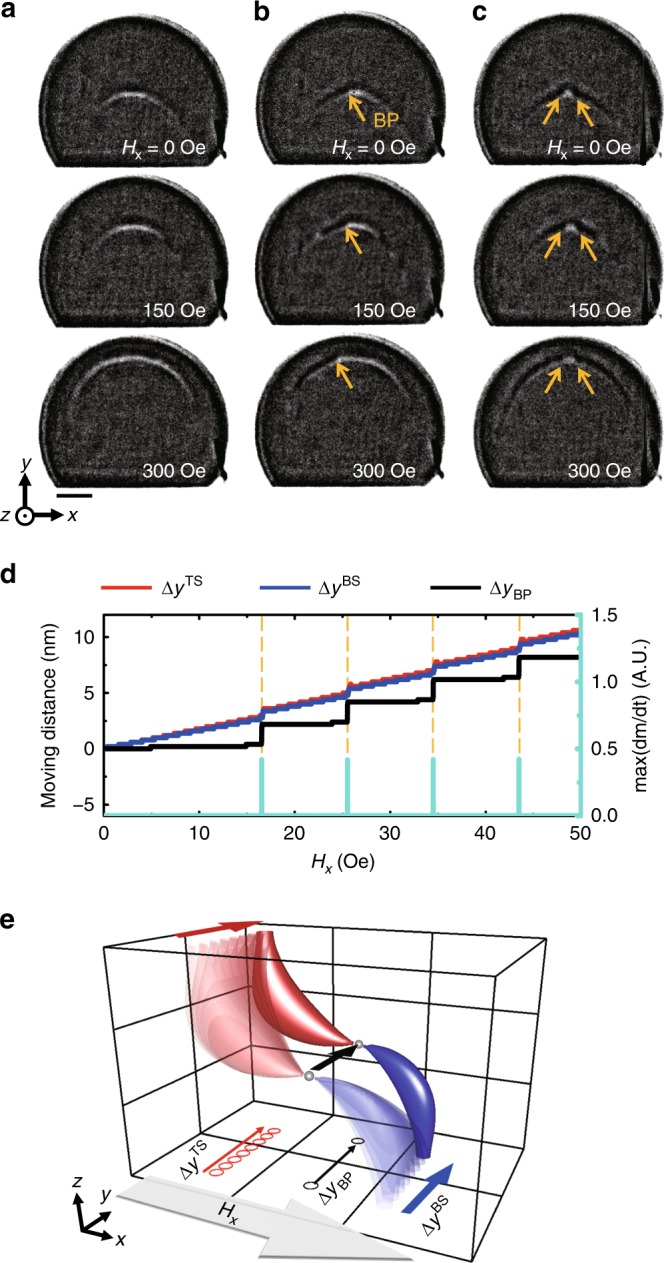
Quasi-static field-driven motions of Bloch points (BPs). **a**–**c** Series of images for BP motions driven by applying an external magnetic field from *H*_*x* *=* _0 to +300 Oe with a field step of 0.5 Oe/ns in the +*x-*direction observed in the non-BP (**a**), single-BP (**b**), double-BP (**c**) cores at *H*_*x*_ = 0, 150, and 300 Oe (scale bar: 500 nm). Damping constant was chosen as α = 0.5 to observe the quasi-static motion of the BP structure as the external magnetic field was slowly increased in the experiment to allow the system to maintain equilibrium. **d** Relative movements of the vortex cores on the top surface (Δ*y*^TS^, red line) and bottom surface (Δ*y*^BS^, blue line) of the disk, and of the BP (Δ*y*_BP_, black line) along the *y*-axis together with maximum rates of d**m***/*dt (turquoise line) obtained by the cubic spline interpolation. **e** Perspective sketch of the quasi-static motions of the vortex cores and BP at the length scale of 0.2 nm

To examine the stability of the observed BPs, we measured the field-driven motions of not only the non-BP core (Fig. 3a), but also those of the single-BP (Fig. 3b) and double-BP (Fig. 3c) cores surrounded by CCW circular domain. By sweeping the external magnetic field from *H*_*x*_ = 0 Oe to + 300 Oe in the +*x*-direction, the BPs move significantly toward the round edge of the disk along the +*y*-direction. Nevertheless, they remain between two vortex cores without any sign of being annihilated, which implies that BPs are under stable equilibrium during their field-driven motions. Because BP is the magnetic singularity point at which no local magnetization exists, its motion is likely triggered by the movements of the vortex cores and ABW that embrace the BP, under the forces exerted by the applied magnetic fields. The path of the BP motion is determined by the rotational sense of the circularity or the field direction. We confirmed that a BP moved to the flat edge of the disk with a CW circularity or with the field sequence from *H*_*x*_ = 0 to −300 Oe (see Supplementary Figure 7 and Supplementary Note 5). It is worth noticing that the motions of BPs on *x*-axis are very limited unlike the case of vortex cores travelling significantly on both *x*- and *y*-axis. Additionally, while the magnetic configuration of BPs remains the same during their quasi-static field-driven motions, the structure of ABWs is considerably deformed when they approach the edge of disk (see Supplementary Figure 8 and Supplementary Note 5). There is a big difference among the field-driven motions of BPs and surrounding magnetic structures, vortex cores, and ABWs. Although the field-driven measurement shows that BPs are topologically robust and they have unique behaviour in their quasi-static motions, the intrinsic atomic-scale nature of a BP with respect to its discontinuous spin configuration could not be determined due to the limitation of the spatial resolution of MTXM (~25 nm). To unveil such nature of BP in the present system, we performed micromagnetic simulations of the field-driven motion of the core structure. The simulations were performed using a cell size of 2 × 2 × 2 nm^3^ and the simulated magnetization configurations were processed by the cubic spline interpolation with 0.2 × 0.2 × 0.2 nm^3^ to finely trace the motions of the BP and vortex cores in a single core (type IV). Figure 3d depicts the relative movements of the vortex cores at the top surface (Δ*y*^TS^, red solid line), bottom surface (Δ*y*^BS^, blue solid line), and the motion of the BP (Δ*y*_BP_, black solid line), along the *y*-axis. The maximum rates of the magnetization change d**m***/*dt are also shown in the graph (turquoise solid line). It can be seen that the BP exhibits a series of jump-like 2 nm displacement motions, and each jump is accompanied by a substantial change in the magnetization. We verified that the same type of jump-like movement with 2 nm step size is observed in the motion of the BP driven by magnetic fields applied in opposite direction, −*x*-direction (Supplementary Figure 9 and Supplementary Note 5). The 2 nm jump size is originated from the cell size of 2 × 2 × 2 nm^3^ used in the simulations where the cells simulate the atomic lattices in a real crystal system. We have chosen 2 nm cell size on the basis that 2 nm presents a reasonable resolution in micromagnetic simulations of BPs to investigate their physical behaviours in the previous theoretical literatures on BP studies^9,10^.

Conversely, the vortex cores move smoothly and continuously. The results in Fig. 3d suggest that the BP within a vortex core structure is pinned by a periodic potential generated at the lattice at approximately every 2 nm, which is comparable to the dimension of a unit cell in the simulation. The BP is then depinned when the externally applied field is sufficiently strong to overcome the energy barrier between lattice points (Fig. 3e). This pinning and depinning behaviour of the BPs at the lattices have been deduced to be caused by their intrinsic nature as discontinuous spin textures^10,15^. The BP enclosed within a vortex core of the present asymmetric disk has relatively lower depinning field (*H*_dpin_ < 17 Oe) than the values calculated in most studies for BPs within DWs of cylindrical magnetic wires (20 Oe *<* *H*_dpin_ < 320 Oe)^10,14,17^ except for the estimated depinning field of 5 Oe reported in the work^18^. This implies that the forces exerted on the BP by the vortex cores and ABW are stronger than those exerted by the DWs in a magnetic nanotube for a given field strength, and therefore the depinning field required to overcome the energy barrier is lower in the present system.

### Dynamics of the single-BP core

**Fig. 4:**
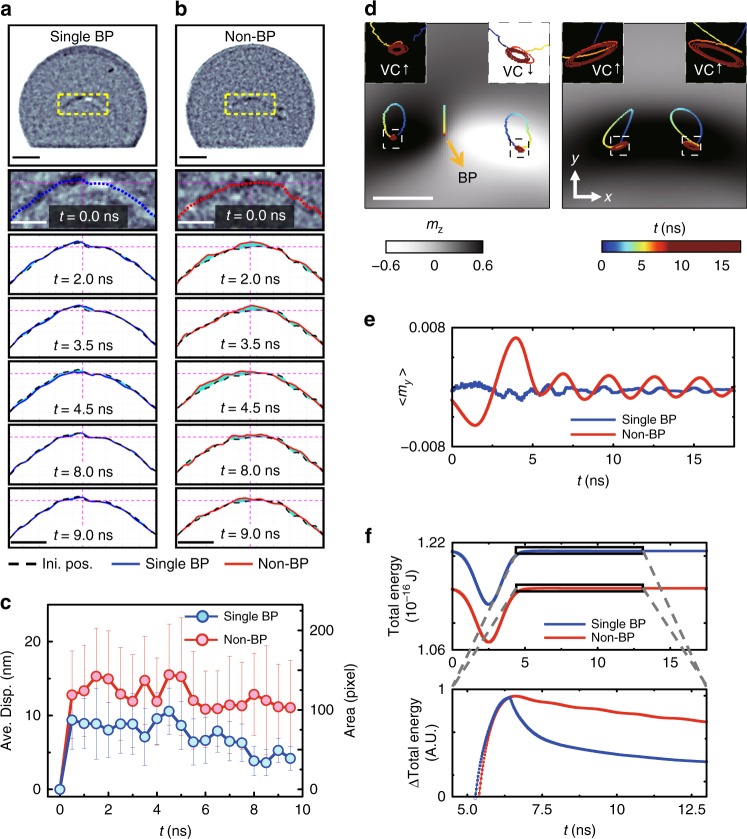
Time-resolved measurements for the dynamics of a Bloch point (BP). **a**, **b** Representative images of the vortex core structures with (**a**) and without (**b**) a BP (the first row) along with positions of core structures measured at *t* = 0, 2, 3.5, 4.5, 8, 9 ns during their dynamic processes triggered by the injection of a field pulse with an amplitude of 50 Oe and the width of 3 ns in the +*x*-direction. The blue and red dotted lines in zoomed images correspond to the initial position of the core structures with and without BP. The red and blue lines indicate perturbed positions (*t* > 0) of the single-BP core and non-BP core. Scale bars in (**a**, **b**) correspond to 500 nm (representative images) and 200 nm (zoomed images), respectively. **c** Quantitatively analysed differences between initial (*t* = 0, black line) and perturbed positions (*t* > 0) of the single-BP core (blue line) and non-BP core structure (red line). The experimental error bars are taken at one standard deviation. **d** Trajectory curves of the simulated dynamic motions of the BP and vortex cores with up (VC^↑^) and down (VC^↓^) polarizations in the single-BP and non-BP cores (scale bar: 50 nm). **e**, **f** The variations of normalized *y*-components of magnetizations, < *m*_*y*_ > = < *M*_*y*_/*M*_*s*_ > (**e**) and total energy (**f**) during the dynamic relaxation processes of the single-BP and non-BP cores. Raw data of Fig. 4c are provided as the Source Data files Source Data

To experimentally prove the nature of a BP, which could not be accomplished by the static measurement of the field-driven motions, we observed and compared the relaxation dynamics of the single-BP core and non-BP core, which would be affected by the energy barriers associated with all lattice points. Figure 4a, b shows representative MTXM images of a single-BP core and a non-BP core, along with positions of core structures measured at *t* = 0, 2, 3.5, 4.5, 8, 9 ns during their dynamic processes triggered by pulse injections (see Supplementary Figure 10, Supplementary Note 6 and Methods). Here, positions of the single-BP and non-BP cores during the dynamic process are indicated by blue and red dotted lines, respectively, and their initial positions at *t* = 0 are represented by black dotted lines. Difference between black and blue (or red) is shaded in turquoise for clear visibility (see Methods).

The dynamic behaviours of the two core structures noticeably differ. The position and shape of the single-BP core marginally varied according to the time, i.e., overlapping of black and blue lines in Fig. 4a, whereas the non-BP core experiences obvious lateral motion and deformation of its shape during the dynamic process, as represented by turquoise solid areas in Fig. 4b. In the movies developed from full image sets taken at intervals of 0.5 ns over 10 ns (see Supplementary Movie 1), the varied dynamic behaviour in the presence of the BP is also obviously observable. We also quantitatively investigated the difference in the dynamic motions of the single and non-BP cores. Figure 4c illustrates quantitative differences in displacements and areas between initial (*t* = 0) and perturbed positions (*t* > 0) of each core. Again, it is clear that the variations of core position and shape during the dynamic process are distinguishably greater in the non-BP core than those of the single-BP core.

The existence of the BP also causes the change in the relaxation mode of the core dynamics. The dynamic motion for the BP core rapidly subsides during the free relaxation with the field pulse turned off, and it is in a sharp contrast to the one of the non-BP core lasting relatively longer. To closely examine the dynamic motions of the BP and vortex cores, micromagnetic simulations for the dynamic processes of those structures were conducted (see Supplementary Figures 12 and Supplementary Note 7). Figure 4d shows the trajectory curves of the dynamic motions of the BP and vortex cores in the single-BP and non-BP cores. As can be seen, the BP travels rectilinearly in the +*y*-direction under pulse injection (0 < *t* < 5 ns), and then returns back to the original position and stays nearby (*t* > 5 ns). Vortex cores, on the other hand, exhibit typical gyroscopic motions, which are observed like slosh rather than gyroscopic with the ABW that connects cores on the top and bottom surfaces (see Supplementary Movie 1).

The different relaxation behaviours of the single-BP and non-BP cores were also verified by the simulation results. As shown in the inserted figures in Fig. 4d, the dynamic motions of the cores that contain the BP attenuate immediately after the first gyration cycle, but the slow relaxation motions of cores with the gradual decrease of oscillation amplitude is witnessed in the non-BP core (see Supplementary Movies 2 and 3, Supplementary Figure 13 and Supplementary Note 7). The rapid relaxation in the BP core is also reflected in the variation of normalized *y*-components of magnetizations, < *m*_*y*_ > and it accompanies a drastic dissipation of the total energy that can be seen in Fig. 4e, f. Same type of dynamic behaviour is also observed in the double-BP core and even it has different magnetic structures around the BPs, two vortex cores with the same polarization, from the one of single-BP core, two vortex cores with opposite polarizations (see Supplementary Figure 14 and Supplementary Note 8). This distinguished dynamic relaxation observed in the presence of BPs can be explained by the fact that a BP experiences energy barriers at lattices with its intrinsic character as a discrete spin texture. After the initial motion of a BP triggered by the application of a field pulse of 50 Oe, the BP tends to remain still in the absence of the supply of further energy to overcome the energy barriers at the lattices. This suppresses the robust and continuous motions of the cores containing the BP. The present experiments thus directly reveal the inherent atomic-scale nature of a BP.

Our results show that a BP can be stably generated in asymmetric ferromagnetic disks and the BP has peculiar dynamic behaviour. We also demonstrate that the existence of a BP considerably acts on the dynamic motion of vortex cores, which is deeply connected to the intrinsic nature of BP as a discrete spin texture. This work paves the way for acquiring BPs readily created, and therefore stimulates further and extensive researches for BPs and their impacts on physical behaviours of other topological spin structures.

## Methods

### Sample fabrication

Polycrystalline Py films were deposited on 100 nm thick X-ray transparent silicon-nitride (Si_3_N_4_) membranes by means of sputtering. The asymmetric disks with a diameter *D* = 2.5 µm, a height *h* = 100 nm, and an asymmetric ratio *r* = 0.2*D* were patterned by electron-beam lithography and lift-off processing.

### Magnetic soft X-ray microscopy measurements

Magnetic structures in Py disks were directly observed utilizing full-field MTXM at the Advanced Light Source (XM-1, beamline 6.1.2)^31^. Magnetic contrast at MTXM is given by X-ray magnetic circular dichroism. The magnetic imaging in Py disks was performed at the Fe L_3_ X-ray absorption edge (708 eV) and IP and OOP magnetic components were imaged with samples mounted at 60° and 90° with respect to the X-ray propagation direction, respectively. To obtain clear magnetic images of IP and OOP components without non-magnetic background, target images were normalized by a reference image taken at saturated state (IP) or an image with opposite photon helicity (OOP). The time-resolved X-ray microscopy for the dynamics of BP core structures was performed at two-bunch mode of the Advanced Light Source in which X-ray photon flashes with the frequency of 3.33 MHz (details in Supplementary Figure 10 and Supplementary Note 6).

### Micromagnetic simulations

Micromagnetic simulations for BP structure and their dynamic excitations were carried out using the MuMax^3^ code^42^ that can solve numerically the Landau–Lifshitz–Gilbert equation: ∂**M**/∂*t* = −*γμ*_0_(**M**×**H**_eff_) + (*α*/|**M**|)(**M** × ∂**M**/∂*t*) with the local magnetization vector **M**, the gyromagnetic ratio *γ*, the effective field **H**_eff_, and the phenomenological damping constant *α*^43,44^. For the simulations we used the mesh size of 2 × 2 × 2 nm^3^ and typical material parameters of Py, i.e., saturation magnetization *M*_s_ = 800 kA/m, exchange stiffness *A*_ex_ = 13 pJ/m, and damping constant *α* = 0.01 (details in Supplementary Figure 11, 12 and Note 7).

### Image analysis

Aligning the MTXM images with sub-pixel resolution is essential to achieve quantitative analysis, such as single (non-)-BP core positions and their deviations after perturbation. We have developed an iterative registration method with intensity-base automatic image alignments. To set the robust reference image from noise raw data, the MTXM images were first roughly aligned using a phase correlation algorithm only with translations of pixel size^45^. We then averaged the first aligned images, and used the image as reference images for second alignments. From the second alignments, intensity-based image registrations algorithm determines the specific image transformation matrix that is applied to the moving image with bilinear interpolation, and bring the misaligned image (raw images) into alignment with the reference image (average of registered image). The same procedures were repeated until the aligned images were self-consistent. Since the iterative registration only updates the reference image, the registered images for all iterative steps can avoid blur from multiple interpolations.

To capture the positions of both core structures with and without BP, salt-and-pepper noise in the MTXM images was reduced by the non-local-mean-filter^46^, which is an algorithm for resulting in better post-filtering clarity, and preserving details in the raw images compared with local-means. Note that the actual spatial resolution (~25 nm) should be somewhat preserved despite filtering procedures, since the pixel size (~10 nm) in the raw image is smaller than the resolution. We then regrouped the pixels along *y*-directions across the single (non-)-BP core structure to get line-profiles of the magnetic contrast and evaluated the deep (dark contrast from positive *M*_*z*_ components) and peak (bright contrast from negative *M*_*z*_ components) of each line-profile by double Gaussian fit. The pixels with the maximum height from either the deep or the peak were defined as the position of single (non-)-BP core structure (Fig. 4a, b). The defined positions showing huge deviation (more than 10 pixels) from their polynomial fit were filtered out. To defined deviation of the position of the single (non-)-BP core from its initial position (*t* = 0, dotted black lines) after applying the pulse (*t* > 0), the position differences were defined by both the averaged displacements from initial position (red and blue solid lines in Fig. 4a, b) and the areas (turquoise shaded area in Fig. 4a, b) between the initial and perturbed positions. Even though the averaged displacements in Fig. 4c are smaller than the resolution of the MTXM, the deviations of individual pixels are larger than the resolution (see Fig. 4c) and measurable.

## Supplementary information

Supplementary Information

Description of Additional Supplementary Files

Supplementary Movie 1

Supplementary Movie 2

Supplementary Movie 3

Source Data

## Data Availability

The data that support the findings of this study are available from the corresponding author upon reasonable request. The source data for the plot in Fig. 4c are provided as aSource Data file.
