# Supplementary material for: Dynamics of the Bloch point in an asymmetric permalloy disk
Source: Nat Commun. 2019 Feb 5;10:593. doi: 10.1038/s41467-019-08327-6 (PMC6363748; doi:10.1038/s41467-019-08327-6)
Supplement: Supplementary file 1 — Supplementary Information [file 41467_2019_8327_MOESM1_ESM.pdf]

Dynamics of the Bloch point in an asymmetric permalloy disk

Supplementary Information

Im *et al*.

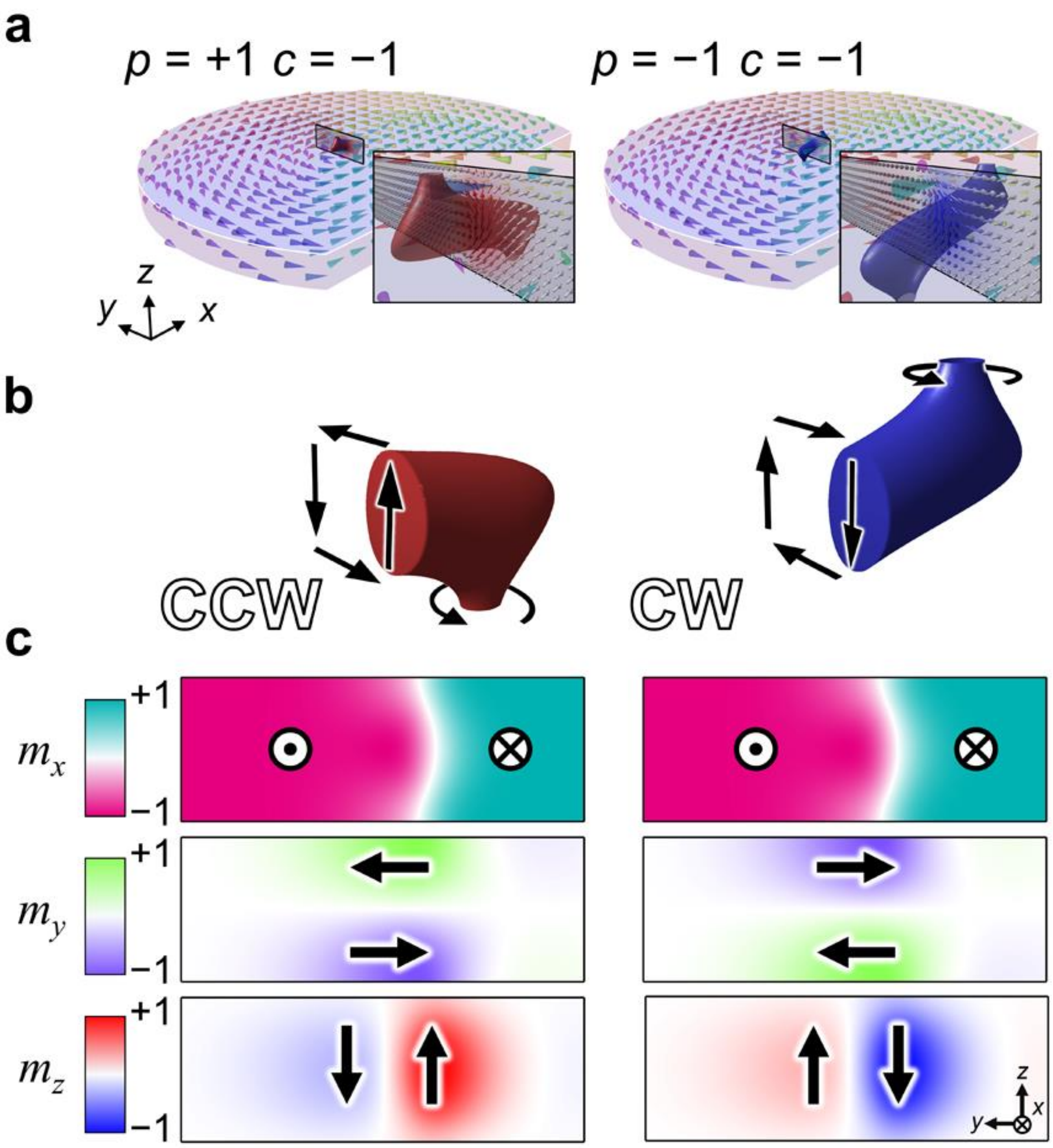

Supplementary Figure 1 | Magnetization configuration of asymmetric Bloch walls. a, Simulated vortex core structures with up and down polarizations, respectively. **b**, The schematic images of CCW and CW flux-closure domain (FCD) structures. **c**, The internal magnetization configurations ($m_x = M_x/M_s$, $m_y = M_y/M_s$, and $m_z = M_z/M_s$) in the cross-section area marked in (**a**).

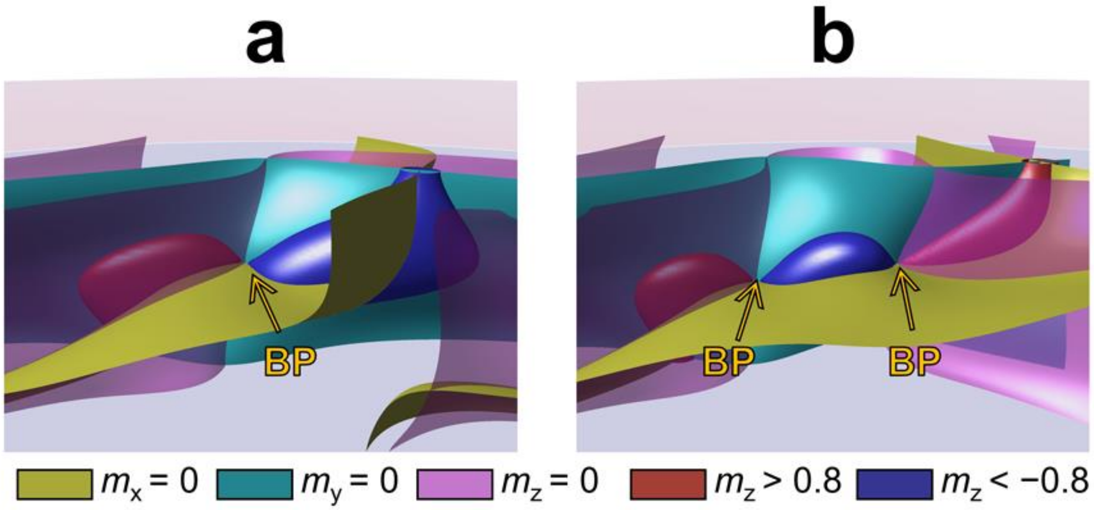

Supplementary Figure 2 | Bloch point within vortex core. The isosurfaces of $m_x = 0$ (yellow), $m_y = 0$ (cyan), and $m_z = 0$ (purple) in a single (**a**) and a double (**b**) BP core structures. The red and blue surfaces correspond to $m_z > 0.8$, and $m_z < -0.8$. The intersection points of those isosurfaces correspond to BPs.

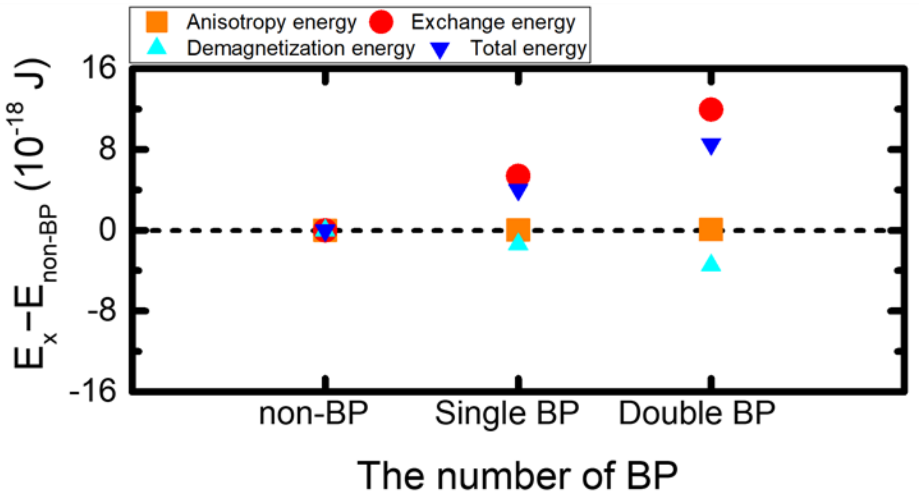

Supplementary Figure 3 | Magnetic energy differences. Differences of magnetic energies between the non-BP core and BP core structures.

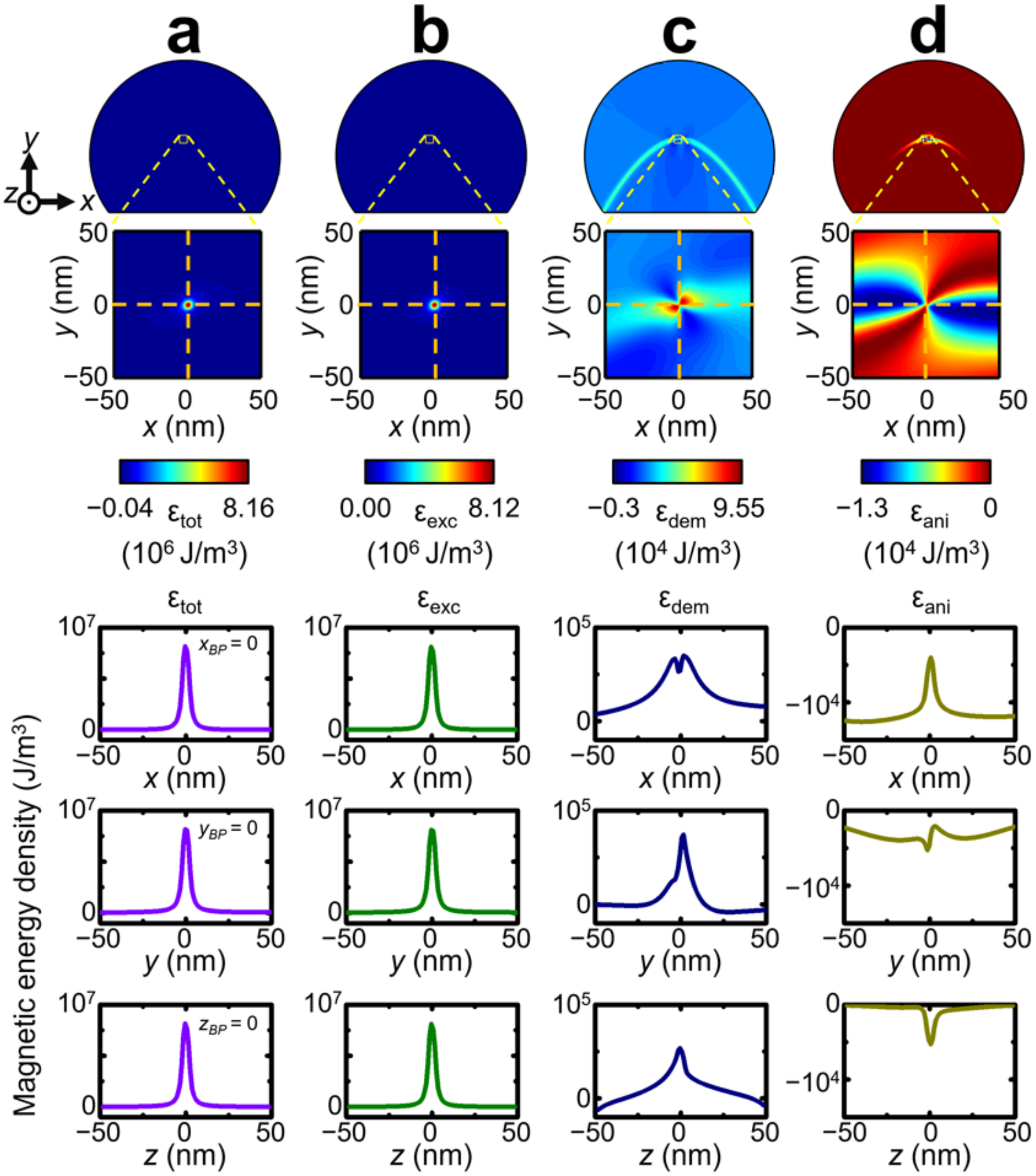

Supplementary Figure 4 | Magnetic energy densities. Spatial distributions of total (**a**), exchange (**b**), demagnetization (**c**), anisotropy (**d**) energy densities in a single BP core together with line profiles of energy densities across the BP on each axis.

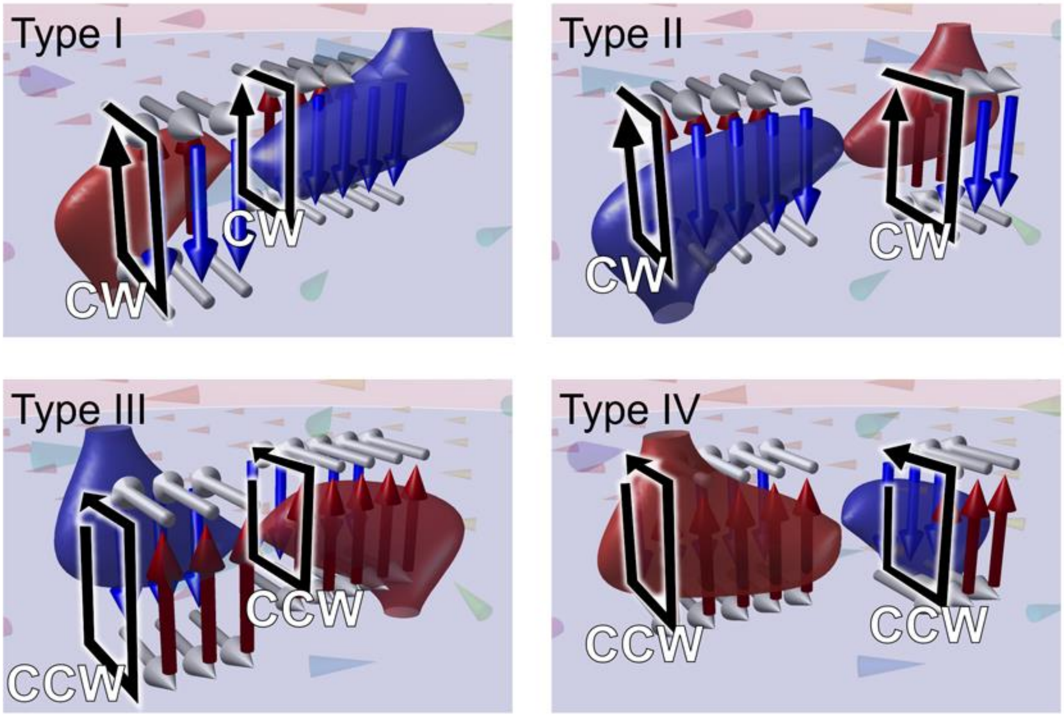

Supplementary Figure 5 | Four different types of single BP core structures. ABW segments with different volumes in the single BP core with $c = -1$. The red and blue colors indicate upward and downward magnetizations, respectively. The orientation of FCD is indicated by arrows. The ABW segment with larger volume is corresponded to the configuration favorably created in the non-BP core.

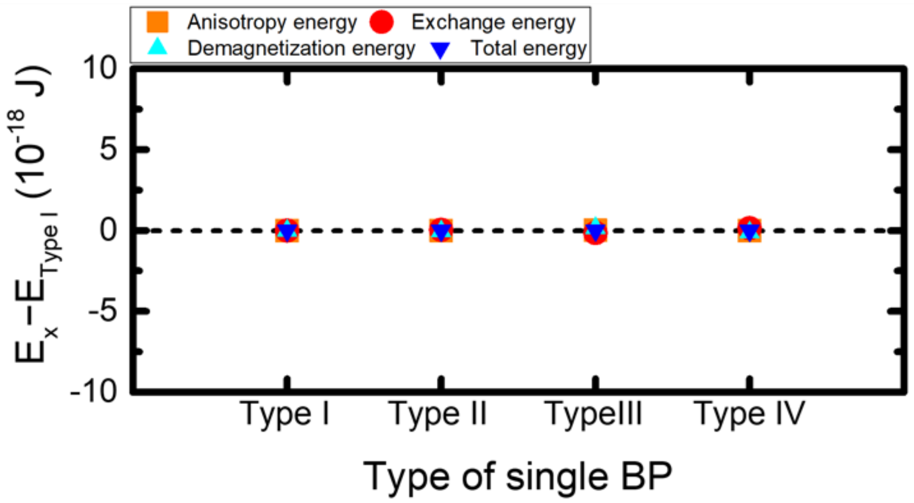

Supplementary Figure 6 | Magnetic energy differences among different types of single BP cores. The magnetic energy differences between four types of the single BP structure and type I of the single BP structure.

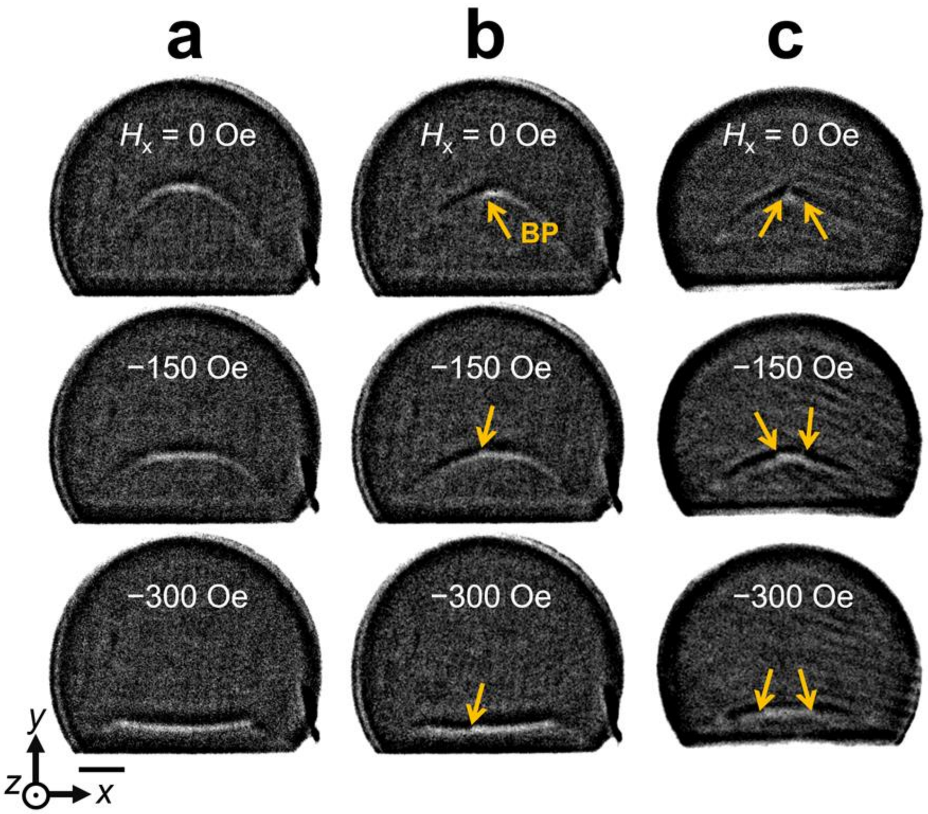

Supplementary Figure 7 | Field-driven motions of the non-BP and BP cores. Series of images for field-driven BP motions observed in a non-BP (**a**), single BP (**b**), and double BP (**c**) core structures with $c = -1$. The black and white contrasts represent the magnetizations oriented in the perpendicular upward and downward to the disk plane. The motions were driven by applying an external magnetic field in the $-x$ direction (H_x) from 0 to –300 Oe. Scale bar corresponds to 500 nm.

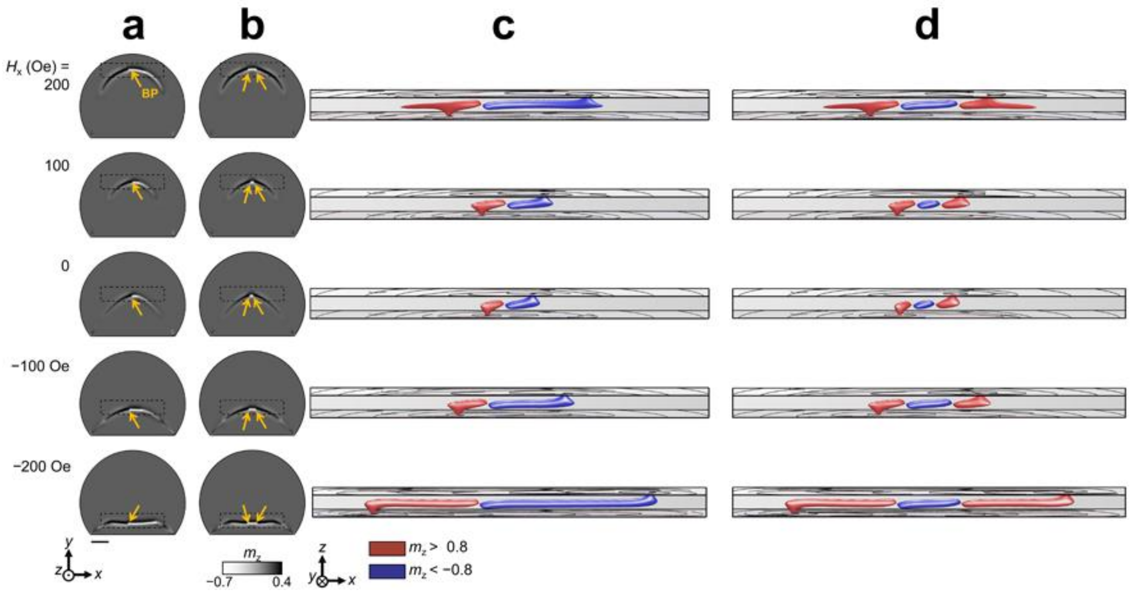

Supplementary Figure 8 | Simulated field driven motions of BP cores. a-b, Simulated images for the field-driven motions of a single (**a**) and double (**b**) BP core structures with $c = -1$ in the field sequences from $H_x = 0$ to +200 Oe and from $H_x = 0$ to –200 Oe in the asymmetric disk with a diameter $D = 2.5$ μm, a height $h = 100$ nm, and an asymmetric ratio $r = 0.2D$. **c-d**, 3D internal magnetic structures of ABW and vortex cores during the field-driven motions of a single (**c**) and double (**d**) BP core structures. Scale bar in **a** corresponds to 500 nm.

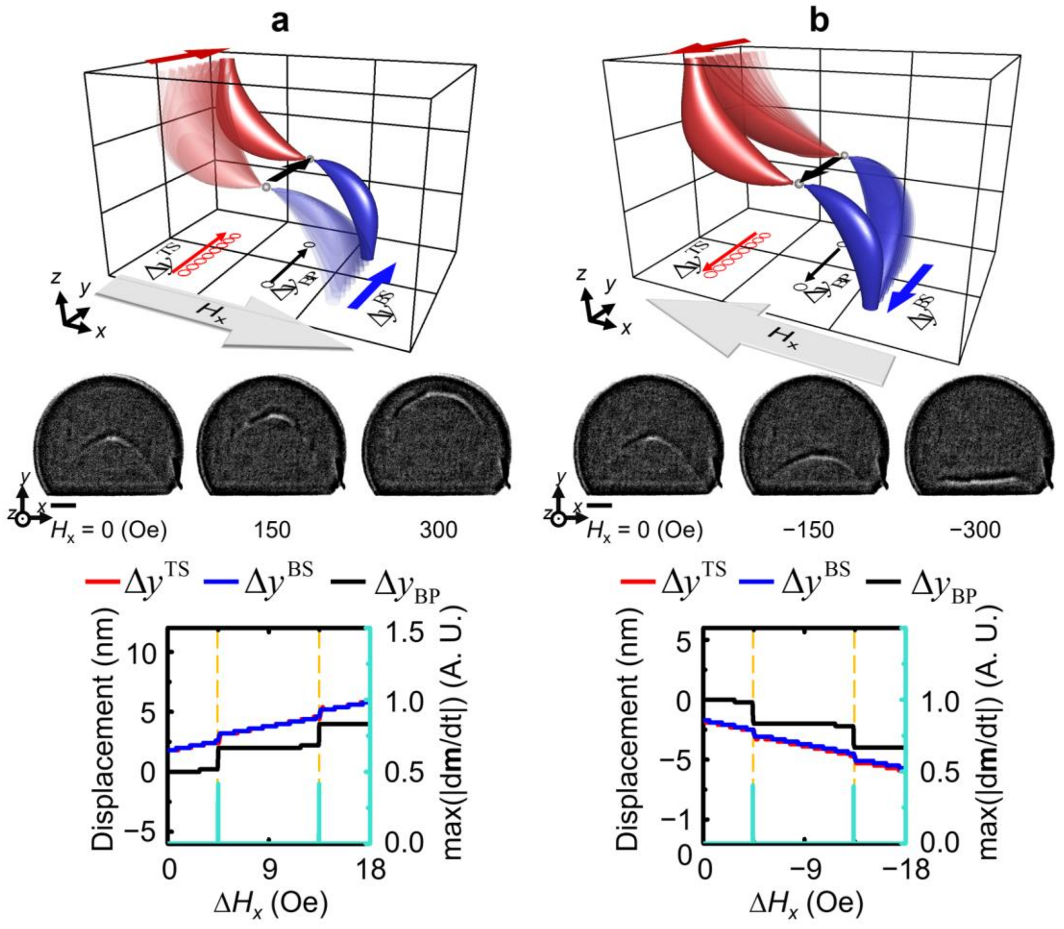

Supplementary Figure 9 | Quasi-static field-driven motions of BP. Perspective sketch of the quasi-static motions of the vortex cores and a BP by applying magnetic field in the positive *x*-direction (**a**) and the negative x-direction (**b**) obtained by micromagnetic simulations performed with the cell size of 2 x 2 x 2 nm^3. Relative movements of the vortex cores on the top surface surface (Δy^{TS}) and bottom surface (Δy^{BS}) of the disk, and of the BP (Δy_{BP}) along the *y*-axis together with the maximum rates of d**m**/dt obtained by micromagnetic simulations are also displayed. Scale bars in **a** and **b** correspond to 500 nm.

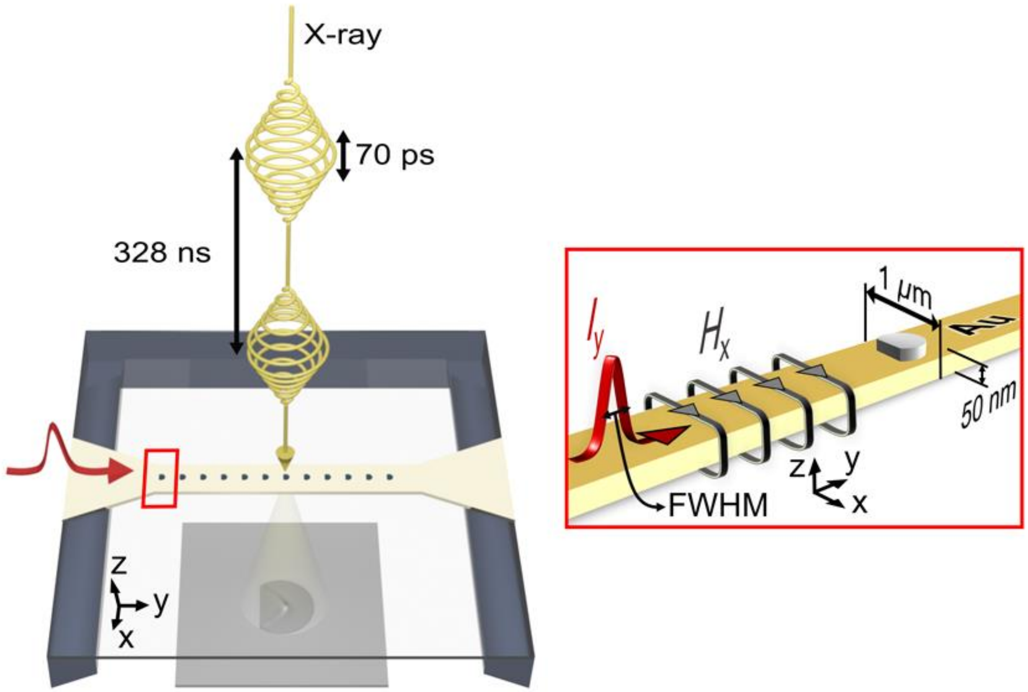

Supplementary Figure 10 | Time-resolved measurement for BP dynamics. Schematic diagram of time-resolved pump-probe X-ray microscopy. The shape of field pulse and the design of electrode are inserted.

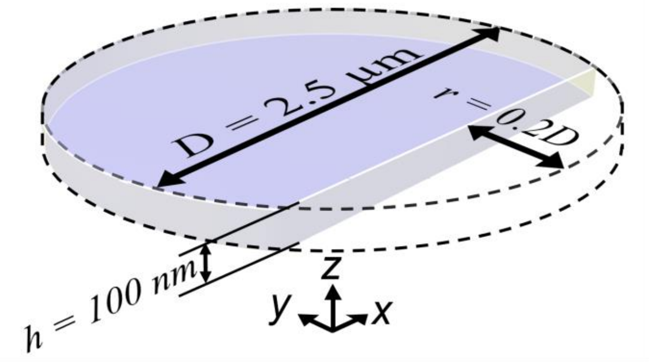

Supplementary Figure 11 | Geometry of the disk for micromagnetic simulations. An asymmetric disk with a diameter D = 2.5 μm, a height h = 100 nm, and an asymmetric ratio r = 0.2D used for micromagnetic simulations.

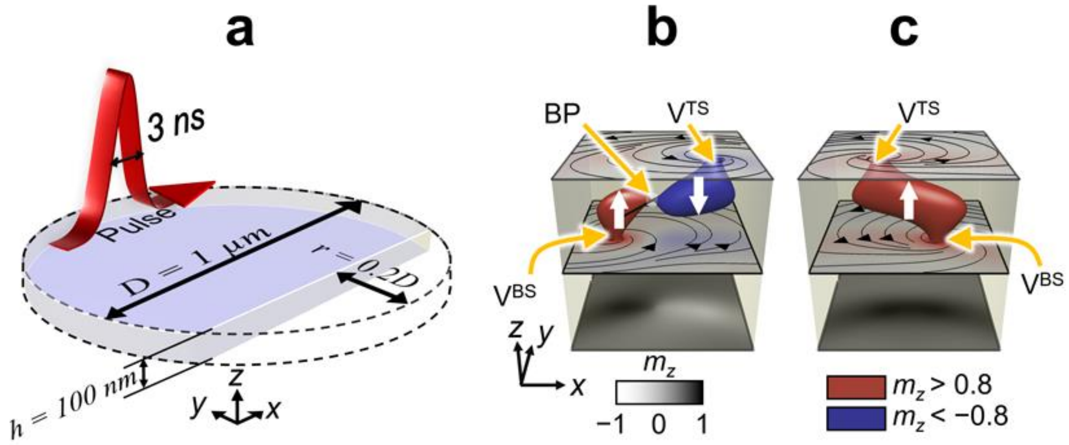

Supplementary Figure 12 | Simulations for dynamics of the non-BP and single BP core. An asymmetric disk of a diameter D = 1 μm, a height h = 100 nm, an asymmetric ration r = 0.2D used for dynamic simulations (**a**) and simulated single BP (**b**) and non-BP (**c**) core structures.

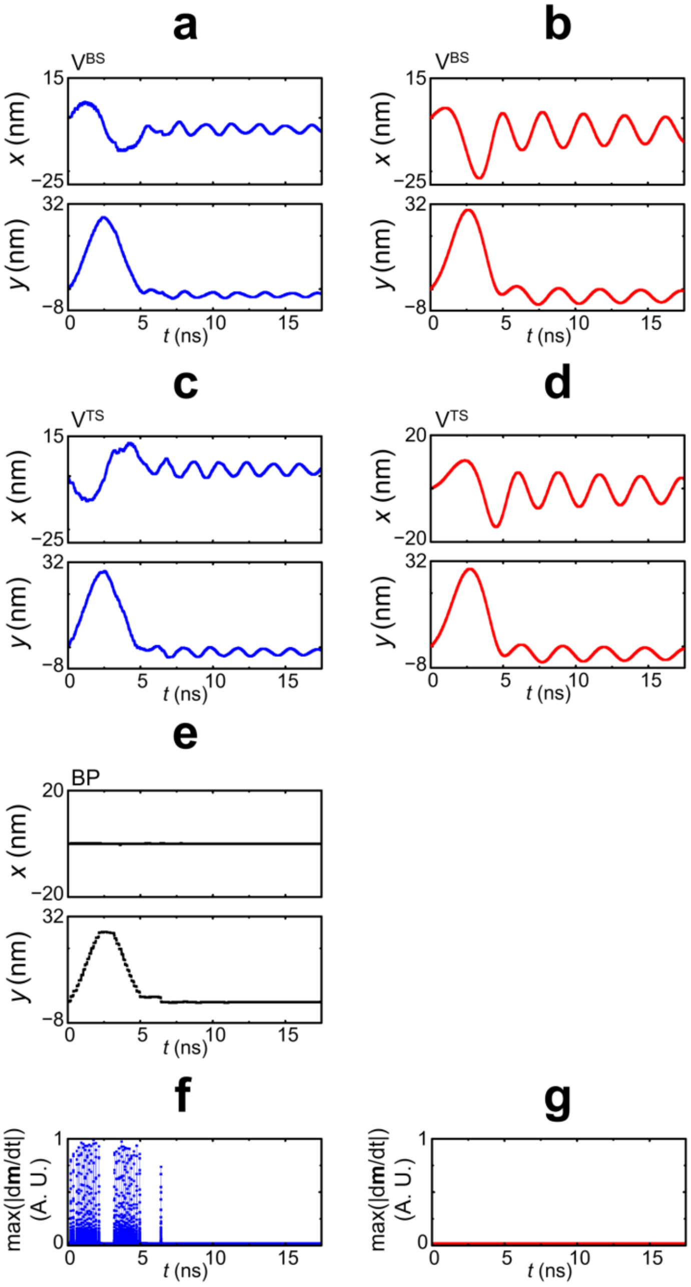

Supplementary Figure 13 | Dynamic motions of vortex cores and BP.| a-e, Displacements of vortex cores on the BS (V^{BS}) (**a-b**) and the TS (V^{TS}) (**c-d**), and the BP (**e**) on *x*- and *y*-axis during the dynamic processes of the single BP core (**a, c, e**) and non-BP core (**b**, **d**). **f-g**, The maximum rates of magnetization change, d**m**/dt during the dynamic processes in the single BP (**f**) core and non-BP core (**g**).

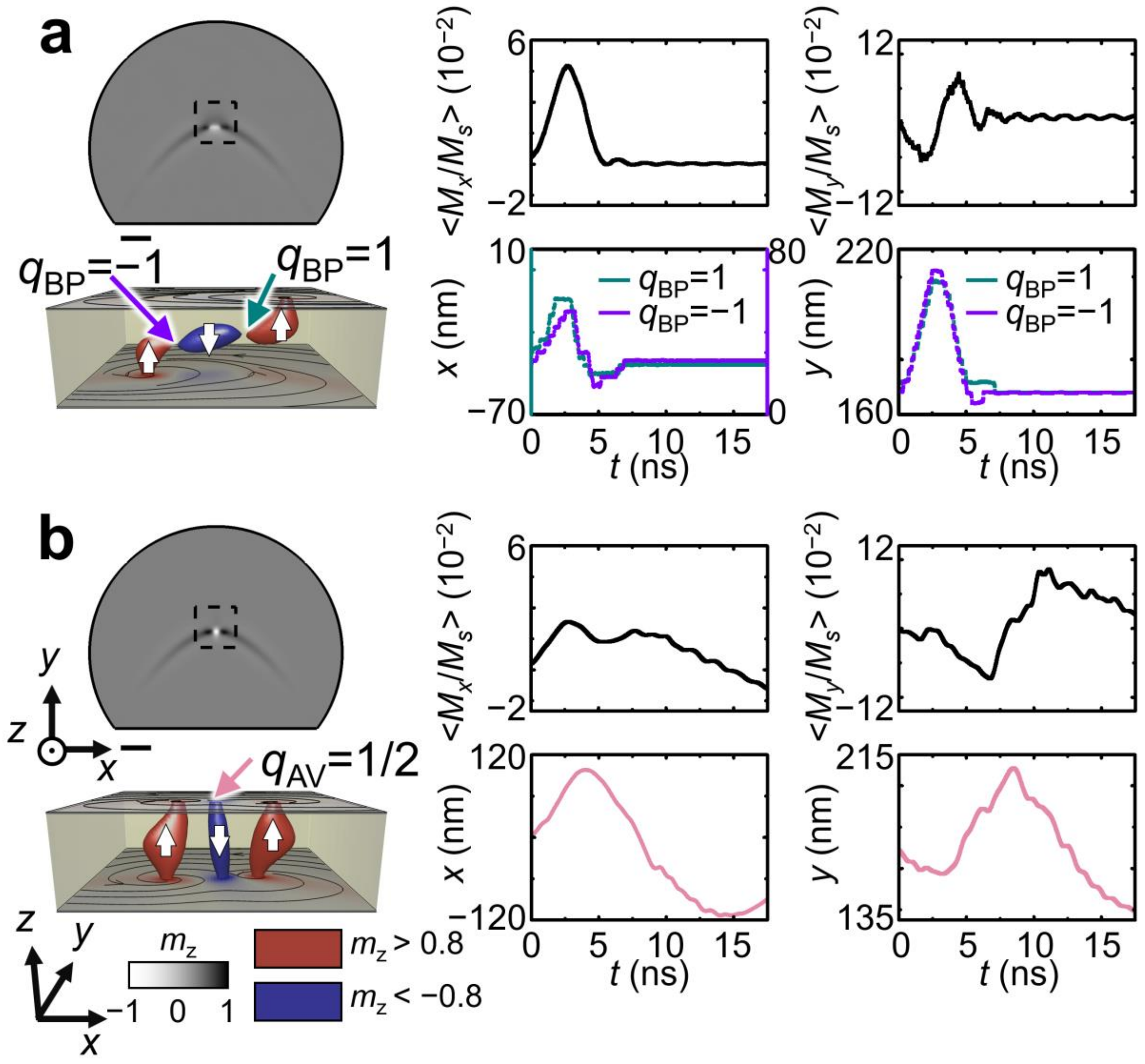

Supplementary Figure 14 | Dynamic motions of a double BP core and vortex-antivortex-vortex (V-AV-V) core structure. The variations of normalized *x*- and *y*-components of magnetizations, <M_x/M_s> and <M_y/M_s> (the first row) and the lateral displacements of BPs and an AV (the second row) during the dynamic relaxation processes of the double BP core (**a**), the V-AV-V structure (**b**). Scale bars in **a** and **b** correspond to 200 nm.

Energy ($\times 10^{-16}$J)	Non-BP core	A single BP core	A double BP core
Anisotropy	–0.131	–0.131	–0.131
Exchange	1.56	1.62	1.68
Demagnetization	1.06	1.04	1.02
Total	2.48	2.52	2.57

Supplementary Table 1 | Magnetic energies for a non-BP, a single BP, a double BP core.

Energy ($\times 10^{-16}$J)	Type I	Type II	Type III	Type IV
Anisotropy	–0.1311	–0.1311	–0.1310	–0.1313
Exchange	1.6172	1.6176	1.6157	1.6186
Demagnetization	1.0434	1.0429	1.0449	1.0421
Total	2.5295	2.5294	2.5296	2.5295

Supplementary Table 2 | Magnetic energies for the type I, type II, type III, and type IV of a single BP core.

Supplementary Note 1. Detailed magnetization configurations of asymmetric Bloch walls

Supplementary Figure 1a shows vortex core structures with up-core polarization (*p* = +1) and down-core polarization (*p* = −1) in which the circular domain rotates counterclockwise (CCW, *c* = −1). The detailed magnetic configuration of the cross-section area inserted in Supplementary Figure 1a was investigated. It turned out that the tube-shaped magnetic segment connected to vortex cores on top and bottom surfaces consists of in-plane (IP) Néel magnetic components on top and bottom surfaces and out-of-plane (OOP) Bloch component in the middle part of the disk, which is referred to as an asymmetric Bloch wall (ABW)[1]. The ABW forms a flux-closer domain (FCD) through the *y*-*z* plane to reduce demagnetization energy at which the Néel caps on top and bottom surfaces point into opposite directions as depicted in Supplementary Figure 1b. The distributions of normalized magnetic components on each axis (m_x, m_y, and m_z) are displayed in Supplementary Figure 1c.

One interesting feature what we found from the simulations was that in the asymmetric disk, FCD always tends to be formed toward the round edge (+*y*-direction) of the disk and, in consequence, only CCW (CW) FCD creates for the ABW with up (down) polarization (see Supplementary Figure 1b). This is clearly distinguished from the case of FCD formed in symmetrically shaped magnetic elements where both CCW and CW FCDs are allowed to be formed regardless of ABW' polarization[2]. More interestingly, each FCD favors a certain type of core configuration. In the vortex structure with *c* = −1, CCW (CW) FCD prefers to be with cores on top-left (top-right) and bottom-right (bottom-left) surfaces (see Supplementary Figure 1a, b).

Supplementary Note 2. Definition of Bloch point (BP) position

Supplementary Figure 2 illustrates the isosurfaces of $m_x = 0$, $m_y = 0$, and $m_z = 0$. The point where the orientation of core polarization switches was found to be the intersection point among those isosurfaces where $m_x = 0$, $m_y = 0$, $m_z = 0$, i.e. a Bloch point.

Supplementary Note 3. Magnetic energies for various core structures

The anisotropy, exchange, demagnetization, and total energies for three core structures are listed in Supplementary Table 1. While the demagnetization energy is diminished by the creation of BPs, the exchange energy is increased as a single and double BPs create. Consequentially, the energy of a non-BP core is lower than those of BP core structures, which implies that BP cores are metastable states. Nevertheless, all three core structures are observed in experiments, which might be due to their subtle energy differences and/or extrinsic factors such as roughness and artificial defects that inevitably exist in real samples. Supplementary Figure 3 shows the differences of magnetic energies between the non-BP core and BP core structures.

Supplementary Figure 4 shows the spatial distributions of magnetic energy densities for the single BP core. Energy densities are maximized at the BP and the exchange energy density particularly undergoes huge spatial variations in the vicinity of the BP.

Supplementary Note 4. Asymmetric volume of up- and down-cores in a single BP core

As discussed in Supplementary Note 1, in an asymmetric disk, there is a symmetry breaking in the formation of FCDs and it leads to the creation of a certain type of core configuration, e.g. the configuration consisting of cores on top-left and bottom-right surfaces is energetically favored to be with CCW FCD. In a single BP core, energetically favored and unflavored configurations within the non-BP core coexist. In the vortex structure with CCW ($c = -1$) circularity, down (up) ABW segment connected to the core on top-right (bottom-left) surface forming CW (CW) FCD is the state corresponded to the energetically favored (unfavoured) configuration in the non-BP core (Type I in Supplementary Figure 5). The volume of energetically favored ABW segment found to be larger than the other and the BP is pushed toward smaller ABW part, which allow us to categorize the type of single BP core structures observed in experiments. The anisotropy, exchange, demagnetization, and total energies for four types of a single circulating BP structure are listed in Supplementary Table 2. The total magnetic energies of the four types of structures are almost identical with very small (~10^{-20} J) differences, which can be considered as the computational errors. Consequentially, it is estimated that four types of the single BP structure are energetically degenerated. Supplementary Figure 6 shows differences of magnetic energies between the type I of the single BP structure and three other types of the single BP structure.

Supplementary Note 5. Quasi-static field-driven motion of Bloch points

Supplementary Figure 7 shows the field-driven motions of BPs when the external field H_x sweeps from 0 to −300 Oe. BPs remain stable while they move toward the flat edge of the disk. Such topologically protected behaviours of BPs were also confirmed by micromagnetic simulations. Even though the two vortex cores on top and bottom surfaces get away from each other and ABW segments are considerably deformed as magnetization reversal proceeds, BPs stay stable and their positions on *x*-axis are not drastically changed (Supplementary Figure 8). The jump-like motion of a BP is revealed regardless of the sign of H_x (Supplementary Figure 9).

Supplementary Note 6. Time-resolved X-ray imaging

To apply magnetic field pulse through the asymmetric disks, Au electrodes with 50 nm thickness and 10 μm width were patterned above the disks. Magnetization dynamics in the asymmetric disk was excited by injecting Gaussian field pulses with the amplitude of V_{pp} = 4V (~50 Oe) and the width (FWHM) of σ = 3 ns (Supplementary Figure 10). The images were acquired with varying the delay time between pump pulse (field pulse) and probe pulse (X-ray pulse) from 0 to 18 ns with an interval of 0.5 ns.

Supplementary Note 7. Micromagnetic simulations for Bloch point structures and their dynamics

To reproduce the BP structures observed in experiments, a certain **M** configuration was initialized and then it was relaxed to local energy minimum **M** states with BPs. The model system (D = 2.5 μm, h = 100 nm, r = 0.2D) used in micromagnetic simulations is shown in Supplementary Figure 11.

To simulate the dynamics of core structures observed in time-resolved experiments, field pulses with the amplitude of V_{pp} = 4V (~50 Oe) and the width of σ = 3 ns were injected in trigger dynamic process of core structures. To reduce computation time while obtaining reliable results, the asymmetric disk of a diameter D = 1 μm, a height h = 100 nm, and an asymmetric ration r = 0.2D was chosen as a model system (see Supplementary Figure 12a). Despite of the smaller size of disk, core structures of the single BP core (Type I) and the non-BP core, which are identical with those observed in experiments, were successfully reproduced (see Supplementary Figure 12b-c).

The dynamic motions of vortex cores on the BS (V^{BS}) and the TS (V^{TS}) in the single BP core and the non-BP core (see Supplementary Figure 13) are investigated by micromagnetic simulations. Supplementary Figure 13a-e illustrates the displacements of vortex cores and the BP on x- and y-axis during the dynamic process. The BP shows the linear motion on y-axis and once it returns to the initial position it doesn't move anymore. More interestingly, the dynamic behavior of vortex cores is considerably changed in the presence of BP. The motions of cores in the BP core structure rapidly subsides, whereas slow relaxation dynamic motion of cores with gradual decrease of amplitude is observed in the non-BP core. The critical role of BP in vortex core dynamics is also witnessed in the variation of the maximum rates of magnetization change,

d**m**/dt during the dynamic process that can be seen in Supplementary Figure 13f-g. The distinguished dynamic relaxations observed in between the single BP and the non-BP cores could be interpreted as a consequence of BP nature associated with its discontinuous spin configuration.

Supplementary Note 8. Dynamic behaviors of the double BP core and vortex-antivortex-vortex core

To confirm that the difference in dynamic behaviors of the BP core and non-BP core what we observed in our work is due to the existence of the BP rather than the different magnetizations of surrounding structures, we additionally performed micromagnetic simulations for the dynamic process of various core structures. Supplementary Figure 14a and b show a double BP core structure and a core structure having the exact same magnetic configuration with the double BP core, two vortex cores with the same up-polarization, respectively. However, in the core structure displayed in Supplementary Figure 14b, the point where the orientation of magnetization switches is an anti-vortex (hereafter, called V-AV-V core) not the BP unlike the case of the double BP core including two BPs. The dynamic behaviors of the double BP core and the V-AV-V core are significantly different although the surrounding magnetic structures are almost identical. To clearly visualize the difference in dynamics of the two core structures, we added the variations of normalized *x*- and *y*-components of magnetizations, $\langle M_x/M_s \rangle$ and $\langle M_y/M_s \rangle$ during the dynamic relaxations. The dynamic relaxation subsides abruptly and almost no dynamic movement occurs after 8 ns in the double BP core. As explained in the manuscript, the rapid relaxation observed in BP cores is likely due to the nature of BP requiring external energy to move in between lattice sites (cells in micromagnetic simulations). Unless the externally supplied energy is strong enough or continuous, BPs tend to stay still at lattice sites (see the plot for the displacement of BPs on *x*- and *y*- axis) and it suppresses dynamic motions of vortex cores. On the other hand, complicate dynamic relaxation lingered long time over the entire dynamic process is observed in V-AV-V core with the AV moving substantially on *x*- and *y*- axis. The dynamics of the V-AV-V core is closer to the dynamic behavior observed in the

non-BP core than the dynamics observed in the double BP core. The results taken from our additional simulations strongly support that the difference between the dynamic responses of the structures with BPs and without BPs are attributed to the BPs presence, and the results confirmed again the significant role of BPs during the dynamic process of vortex core structures.

Supplementary References

1. A. Hubert, R. Schäfer, *Magnetic Domains* (Springer, Berlin, 1998).

2. Hertel, R. Kronmüller, H. Computation of the magnetic domain structure in bulk permalloy. *Phys. Rev. B* **60**, 7366-7378 (1999).
