# Supplementary material for: Dynamics of the Bloch point in an asymmetric permalloy disk
Source: Nat Commun. 2019 Feb 5;10:593. doi: 10.1038/s41467-019-08327-6 (PMC6363748; doi:10.1038/s41467-019-08327-6)
Supplement: Supplementary file 2 — Description of Additional Supplementary Files [file 41467_2019_8327_MOESM2_ESM.pdf]

Description of Additional Supplementary Files

Supplementary Movie 1 | Dynamic motions of the single BP and non-BP cores

Movies are full image sets taken from time-resolved X-ray microscopy in the single BP and non-BP cores. The images were acquired with an interval of 0.5 ns over 10 ns while a Gaussian field pulse with the amplitude of V_{pp} = 4V (~50 Oe) and the width (FWHM) of σ = 3 ns was applied to the disk.

Supplementary Movies 2 and 3 | Simulations on dynamic motions of the single BP and non-BP cores

Movies 2 and 3 are full sets of simulated images for the dynamic motions of vortex cores and the BP triggered by injecting field pulse with the amplitude of V_{pp} = 4V and the width of σ = 3 ns in the single BP and non-BP cores. The displacements of vortex cores and the BP during the dynamic process on the *x*- and *y*-axis are also added.
